# The Roles of the Gut Microbiota and Chronic Low-Grade Inflammation in Older Adults With Frailty

**DOI:** 10.3389/fcimb.2021.675414

**Published:** 2021-07-01

**Authors:** YuShuang Xu, XiangJie Liu, XiaoXia Liu, Di Chen, MengMeng Wang, Xin Jiang, ZhiFan Xiong

**Affiliations:** ^1^ Division of Gastroenterology, Liyuan Hospital, Tongji Medical College, Huazhong University of Science and Technology, Wuhan, China; ^2^ Institute of Geriatric Medicine, Liyuan Hospital, Tongji Medical College, Huazhong University of Science and Technology, Wuhan, China; ^3^ Division of Geriatric, Liyuan Hospital, Tongji Medical College, Huazhong University of Science and Technology, Wuhan, China

**Keywords:** gut microbiota, chronic low-grade inflammation, aging, frailty, review

## Abstract

Frailty is a major public issue that affects the physical health and quality of life of older adults, especially as the population ages. Chronic low-grade inflammation has been speculated to accelerate the aging process as well as the development of age-related diseases such as frailty. Intestinal homeostasis plays a crucial role in healthy aging. The interaction between the microbiome and the host regulates the inflammatory response. Emerging evidence indicates that in older adults with frailty, the diversity and composition structure of gut microbiota are altered. Age-associated changes in gut microbiota composition and in their metabolites contribute to increased gut permeability and imbalances in immune function. In this review, we aim to: identify gut microbiota changes in the aging and frail populations; summarize the role of chronic low-grade inflammation in the development of frailty; and outline how gut microbiota may be related to the pathogenesis of frailty, more specifically, in the regulation of gut-derived chronic inflammation. Although additional research is needed, the regulation of gut microbiota may represent a safe, easy, and inexpensive intervention to counteract the chronic inflammation leading to frailty.

## Introduction

Frailty is a clinical syndrome characterized by “diminished strength, endurance, and reduced physiological function”. Frailty predisposes patients to negative health-related outcomes such as falls, hospitalization, disability, dependency, and mortality (Hirani et al., 2015; Wilson et al., 2017; Picca et al., 2019). The prevalence of frailty ranges from 4% to 59% in community-dwelling older adults and increases with age (Collard et al., 2012). Given the rapidly aging population, the United Nations estimates that worldwide, the number of people aged 60 years and above will double to nearly 2.1 billion by 2050 (Jayanama and Theou, 2019). Therefore, frailty is a pressing concern in aging societies (Morley et al., 2013).

Aging is an irreversible, complex biological process determined by a combination of genetic, epigenetic, and environmental factors (O’Toole and Jeffery, 2015; Melzer et al., 2020). Microorganisms, as an environmental factor, are among the most interesting contributors to aging, and they provide a new perspective in understanding the aging process (Finlay et al., 2019). As a person ages, progressive changes in intestinal tract physiology, the intestinal mucosal immune system, lifestyle changes (particularly in diet and exercise), medication, malnutrition, inflammation, and immune senescence may change the diversity, composition and functional features of the gut microbiota (Li et al., 2016; Adriansjach et al., 2020; DeJong et al., 2020). Data from animal models demonstrate that age-related microbial dysbiosis contributes to intestinal permeability, systemic inflammation, and premature mortality (DeJong et al., 2020). Though the cause-and-effect relationship is unclear, age-related microbial dysbiosis is linked to unhealthy aging and geriatric syndromes, which include frailty. Identifying specific changes in frailty-related gut microbiota is essential in developing microbiome-based diagnostic and therapeutic strategies.

In this review, we first describe the relevant changes in gut microbiota related to aging and frailty. Subsequently, we summarize recent findings on the possible role of chronic low-grade inflammation in frailty and how microbial dysbiosis is involved in its pathogenesis, including frailty-related inflammation.

## The Definitions and Inflammatory Biomarkers of Frailty

The concept of frailty was formally put forward in the 1970s and since then has continuously evolved (Rockwood K et al., 1994). Frailty is not the result of a singular disease—it often follows from a series of chronic diseases, an acute event, or a serious disease. Frailty usually involves physical and psychosocial factors, which can be subdivided into physical frailty, cognitive frailty, and psychosocial frailty (Malmstrom TK, 2013).

The definition of physical frailty is widely recognized; it manifests as a decrease in physical strength and physiological reserve, and minor external stimuli can cause clinical events. The frailty phenotype (FP) and frailty index (FI) are two leading assessment criteria used to identify physical frailty. According to Fried’s FP, of the five clinical symptoms (unintentional weight loss, loss in grip strength, self-reported exhaustion, slow walking speed, and low physical activity), individuals with three are considered frail, and those with one or two are identified as pre-frail (Cesari et al., 2014). In contrast, the FI is a dynamic, continuous process that involves the accumulation of deficits, and as these deficits increase, frailty increases. The FI may be useful in assessing the effectiveness of interventions as well as tracking any changes or trends in a patient’s health situation (Cesari et al., 2014).

Cognitive frailty is a subtype of frailty (Ruan et al., 2015). It refers to the coexistence of physical frailty and reduced cognitive reserves, which include mild cognitive impairment (MCI) (i.e., Clinical Dementia Rating score equal to 0.5) but not dementia (Ticinesi et al., 2018). Cognitive frailty is considered to be a form of pathological brain aging and is an early sign of neurodegeneration (Ruan et al., 2015). The characteristics of cognitive impairment, such as deterioration of executive function and attention, are related to weak grip strength and slow walking speed (Robertson et al., 2014). In the aging process, cognitive and physical frailty interact and increase the risk of poor prognosis (Robertson et al., 2013). At present, no unified assessment tool for cognitive frailty exists. Instead, assessments of physical frailty are often combined with cognitive function assessment scales to screen older adults with cognitive frailty.

As proposed by Franceschi *et al.* in 2000, “inflamm-aging” is an age-associated proinflammatory status and consists of the reduced capacity to cope with various stressors (Franceschi C and Valensin, 2000). Although its causal relationship is still unclear, the relevance of systemic inflammation to the pathophysiology of frailty has been reported in many papers. The potential inflammatory biomarkers of older adults with frailty are summarized in **Table 1**.

**Table 1:** Potential inflammatory biomarkers of older adults with frailty.

Biomarker	Nature	Diagnostic criteria	Setting	Regulation in frailty	Reference
WBC	Blood cell	FP	Community	Up-regulated	Castellana et al. (2021)
					Fontana et al. (2013)
Lymphocytes	Blood cell	FP	Hospital	Down-regulated	Navarro-Martinez et al. (2019)
					Fontana et al. (2013)
CRP	Protein	FP	Hospital, Community	Up-regulated	Navarro-Martinez et al. (2019)
					Marcos-Perez et al. (2018)
					Fontana et al. (2013)
					Yang et al. (2018)
PCT	Protein	FP	Hospital	Up-regulated	Yang et al. (2018)
IL-1α	Cytokine	FP	Community	Up-regulated	Nascimento et al. (2018)
IL-6	Cytokine	FP, FI	Community	Up-regulated	Castellana et al. (2021)
					Navarro-Martinez et al. (2019)
					Marcos-Perez et al. (2018)
					Nascimento et al. (2018)
					Hsu et al. (2019)
					Qu et al. (2009)
					Yang et al. (2018)
					Rusanova et al. (2018)
IL-8	Cytokine	FP, FI	Hospital, Community	Up-regulated	Navarro-Martinez et al. (2019)
					Hsu et al. (2019)
					Rusanova et al. (2018)
IL-10	Cytokine	FP	Community	Up-regulated	Furtado et al. (2020)
					Rusanova et al. (2018)
TNF-α	Cytokine	FP	Community, Hospital	Up-regulated or Down-regulated	Castellana et al. (2021)
					Furtado et al. (2020)
					Navarro-Martinez et al. (2019)
					Marcos-Perez et al. (2018)
					Nascimento et al. (2018)
					Rusanova et al. (2018)
CXCL10	Chemokine	FP	Community	Up-regulated	Qu et al. (2009)
MCP-1	Chemokine	FP	Community	Up-regulated	Lu et al. (2016)
RANTES	Chemokine	FP	Community	Up-regulated	Lu et al. (2016)
ICAM-1	Protein	FP	Community	Up-regulated	Lee et al. (2016)
sRAGE	Protein	FP	Community	Up-regulated	Butcher et al. (2019)

WBC, White blood cells; PCT, Procalcitonin; IL, Interleukin; TNF-α, Tumor necrosis factor alpha; CXCL10, Chemokine (C-X-C motif) ligand10; FP, Fraity phenotype; FI, Frailty index; MCP-1, Chemokine monocyte chemotactic protein-1; RANTES, Regulated on activation, normal T cell expressed and secreted; ICAM-1, Intercellular adhesive molecule-1; sRAGE, The soluble receptor for advanced glycation end-products.

White blood cells (WBC), lymphocytes, C-reactive protein (CRP), interleukin (IL)-6, IL-10, and tumor necrosis factor alpha (TNF-α) are inflammatory biomarkers that have gained attention in the past decade (Qu et al., 2009; Fontana et al., 2013; Soysal et al., 2016; Marcos-Perez et al., 2018; Nascimento et al., 2018; Rusanova et al., 2018; Yang et al., 2018; Navarro-Martinez et al., 2019; Hsu et al., 2019; Furtado et al., 2020; Castellana et al., 2021). A meta-analysis from 2016 concluded that older adults with frailty and pre-frailty had significantly higher levels of CRP, TNF-α, IL-6, white blood cells, and fibrinogen (Soysal et al., 2016). However, this conclusion was mainly based off of cross-sectional studies; not enough high-quality longitudinal data were available for consistent conclusions to be drawn. Lymphocytes are significantly positively correlated with both physical activity and hand grip strength. Grip strength refers to the maximum grip strength of the dominant hand; it is used as a single marker of frailty in similarly aged people (Fernández-Garrido et al., 2014). IL-10 is a controversial biomarker, with part of the study revealing high serum concentrations of IL-10 in frail groups (Rusanova et al., 2018; Furtado et al., 2020). But differs from the frail humans, the genetically altered IL-10 tm/tm mouse, which lacks IL-10 expression, is more sensitive to the activation of inflammatory pathways (Walston and Yang, 2008).

Hus et al. suggested that frailty may not be a nonspecific pan-inflammatory condition and is cross-sectionally associated with IL-6 and IL-8 (Hsu et al., 2019). IL-1α plasmatic levels in pre-frail subjects are significantly higher than non-frail ones (Nascimento et al., 2018). Yang et al. reported that procalcitonin (PCT) is associated with frailty in a non-infected group rather than the pneumonia group (Yang et al., 2018). (C-X-C motif) ligand (CXCL)10 (CXCL10) is a potent pro-inflammatory chemokine that is increased in frail participants. In turn, frailty-associated CXCL10 upregulation is related to elevated serum IL-6 levels (Qu et al., 2009). Similar to the changes observed during aging, chemokine monocyte chemotactic protein-1 (MCP-1) and regulated on activation, normal T cell expressed and secreted (RANTES) are positively associated with frailty (Lu and Nyunt, 2016). Lee et al. observed higher levels of intercellular adhesive molecule-1 (ICAM-1) in frail older adults and consequently proposed that leukocyte migration and inflammation cascade activation may be involved in the development of frailty (Lee et al., 2016). Finally, soluble receptor for advanced glycation end-products (sRAGE) is associated with a higher risk of death among frail subjects (Butcher et al., 2019).

## Alterations of the Gut Microbiota in Aging and Frailty

### Aging-Related Changes in the Gut Microbiota

Changes in the gut microbiota are closely related to the aging process and its related disease outcomes (Bana and Cabreiro, 2019). Compared to younger adults, the gut microbiota composition in older adults is less diverse, and the inter-individual variation is greater (Claesson et al., 2012; Bischoff, 2016). This larger variation may be related to external factors that influence the gut microbiota such as diet, exercise, or medication (O’Toole and Jeffery, 2015). Claesson et al. analyzed the fecal microbiota of 161 older adults (aged over 65) and 9 younger controls. They found a greater proportion of *Bacteroides* spp. and distinct abundance patterns of Clostridium groups in the fecal microbiota of older adults. The proportions of some disease- or health-related phyla and genera such as *Proteobacteria*, *Actinobacteria*, and *Faecalibacteria* also varied dramatically among the individuals (Claesson et al., 20111). Notably, another study reported that in the same population, the gut microbiota composition of healthy older adults and healthy young participants were similar (Bian et al., 2017). Accordingly, “healthy and younger” gut microbiota may be beneficial for healthy aging.

Longevity is a complex process that involves longer life expectancies and a slower pace of aging. The gut microbiota of Sardinian centenarians display decreases in *Faecalibacterium prausnitzii* and *Eubacterium rectale* and increases in *Methanobrevibacter smithii* and *Bifidobacterium adolescentis* (Wu L et al., 2019). Notably, decreases in F. *prauznitzii* were reported by Biag et al. to have anti-inflammatory properties. They also found that *Eubacterium citrate* and their relatives, considered to be signature bacteria of a longer life, were increased approximately 15-fold in centenarians (Biagi et al., 2010). Moreover, the gut microbiota of centenarians (aged 99–104 years) and semi-supercentenarians (aged 105–109 years) demonstrated an increased abundance of species from *Akkermansia*, *Bifidobacterium*, and *Christensenellaceae* (Biagi et al., 2016).

Although the community compositions and structures of gut microbiota differ between Chinese and Italian centenarians, they do exhibit some common features that are different from younger people. For example, the alpha diversity of the gut microbiota is increased in the long-living group, and *Clostridium* cluster XIVa, *Ruminococcaceae*, *Akkermansia* and *Christensenellaceae* are enriched (Kong et al., 2019). *Clostridium* cluster XIVa and *Ruminococcaceae* are genera that contain butyrate-producing bacteria. Butyrate is an most important energy source of colon epithelial cells and has anti-inflammatory properties (Liu et al., 2018). *Akkermansia muciniphila* is a promising probiotic and is associated with host metabolic functions and immune responses (Dao et al., 2016; Zhang et al., 2019). Finally, the human gut bacteria *Christensenellaceae* is negatively correlated with metabolic disorders and inflammation-related diseases such as inflammatory bowel disease (Waters and Ley, 2019).

### The Gut Microbiota and Frailty

We summarized available articles regarding the relationship between changes in the gut microbiota and frailty in the older adult population. The results are listed in **Table 2**.

**Table 2:** Studies investigating the association between fecal microbiota composition and frailty in older adults.

Study (year)	Country	Setting	Group being compared	Number of participants T(F/C)	Mean age (F/C)	Method of frailty	Mean finding in frailty group	Microbiota profiling
van Tongeren et al. (2005)	Netherlands	Elderly Center	Elderly with a low frailty score VS Elderly with a high frailty score	23 (13/10)	NA/NA	GFI	↑ *Enterobacteriaceae*	Fluorescent in situ hybridization
							↓ *Bacteroides/Prevotella Faecalibacterium prausnitzii*	
Claesson et al. (2012)	Irish	Community, Out-patient day hospital, Shortterm rehabilitation hospital, long-term residential care	Healthy community dwelling subjects VS frail long-term care residents	178 (NA/NA)	78	Weight, CC	↑ *Alistipes, Oscillibacter*	16S rRNA sequencing
							↓ *Ruminococcus, Prevotella*	
Jackson et al. (2016)	UK	Community	Pre-frail VS Non-frail	728 (103/625)	63	FI	Frailty is negatively associated with alpha diversity of the gut microbiota	16S rRNA sequencing
							↑ *Eubacterium dolichum, Eggerthella lenta*	
							↓ *Faecalibacterium prausnitzii*	
Haran et al. (2018)	USA	Nursing home	A prospective longitudinal cohort study	23	82.7	CSHA-CFS	↓ Butyrate-producing organisms	16S rRNA sequencing
Zhang et al. (2020)	China	Hospital	Frailty group VS Control group	27 (15/12)	82.0/81.2	FI	No differences in alpha diversity	16S rRNA sequencing
							↑ *Acetanaerobacterium,Catenibacterium, [Ruminococcus]_torques_group, DQ801572_g, Ruminococcaceae_UCG -011, Prevotella_9, Olsenella, EF434341_g, KF843164_g, Pseudoxanthomonas*	
							↓ *Gemella, Lachnoanaerobaculum, Eubacterium[U1]_ruminantium_group, Azospira, Tyzzerella, Cloacibacterium, EU455341_g genera*	
Picca et al., (2019)	Italy	Community	PF&S VS NonPF&S	35 (18/17)	75.7/73.9	SPPB	No differences in alpha diversity	16S rRNA sequencing
							↑ *Oscillospira, Ruminococcus*	
							↓ *Barnesiellaceae, Christensenellaceae*	
Margiotta et al. (2020)	Italy	Hospital	F-CKD VS NF-CKD	64 (38/26)	81.8/79.0	FFP	No differences in alpha diversity	16S rRNA sequencing
							↑ *Mogibacteriacee, Coriobacteriacee Eggerthella*	
Lim et al. (2021)	Korea	Community	Frail VS Pre-frail VS Robust	176 (8/26/142)	74.7	FI	FI scores were negatively associated with microbial diversity	16S rRNA sequencing
							↑ *Bacteroides fragilis Clostridium hathewayi*	
							↓ *Prevotella copri, Coprococcus eutactus*	

GFI, Groningen Frailty Indicator; CC, Calf circumference; FI, Frailty Index; CSHA-CFS, Canadian Study of Health and Aging’s seven-point clinical frailty scale; F-CKD, CKD patients with frailty; NF-CKD, CKD patients without frailty; FFP, Fried’s Frailty Phenotype; PF&S, Physical frailty and sarcopenia; NonPF&S, Non nonsarcopenic nonfrail; SPPB, Short physical performance battery.

A study from the Heymans Older Adults Center using a small sample evaluated the fecal microbial composition of older adults with frailty and found a significant reduction in the abundance of *Bacteroides/Prevotella* and *F. prausnitzii*. While the numbers of *Enterobacteriaceae* were higher (van Tongeren et al., 2005). *Prevotella* is a common constituent of the human intestinal microbiota, especially in the non-Westernized population. The succinate producer *Prevotella copri* improves glucose and insulin tolerance in people with fiber-rich diets, suggesting that the beneficial effects of *Prevotella* may be diet-dependent (Tett et al., 2019). *F. prausnitzii* is an important butyrate-producing bacteria with anti-inflammatory properties (Ferreira-Halder et al., 2017). In contrast, some forms of *Enterobacteriaceae* are opportunistic pathogens that can cause intestinal and extraintestinal infections (Sassone-Corsi et al., 2016).

The loss of community-associated microbiota is associated with increased frailty. Decreases in the numbers of taxa such as *Ruminococcus* and *Prevotella*, along with increases in the numbers of *Alistipes* and *Oscillibacter*, are correlated with several parameters of frailty (Claesson et al., 2012). These results suggest that the number of butyrate-producing genera is decreased, while the number of genera that can metabolize fermented products is increased. Butyrate helps suppress colonic inflammation and carcinogenesis (Fu et al., 2019), and it can directly acylate virulence factors to reduce the invasion and infection ability of pathogens such as *Salmonella typhimurium* (Michaudel and Sokol, 2020). In contrast, *Alistipes* is pathogenic in colorectal cancer and is related to mood disorders (Parker et al., 2020).

Jackson et al. reported a negative relationship between frailty and the alpha diversity of gut microbiota. In pre-frail individuals, *Eubacterium dolichum* and *Eggerthella lenta* are more abundant, while *F. prausnitzii* is less so (Jackson et al., 2016). *E. lenta*, which belongs to the *Actinobacteria* phylum, is associated with inflammation (Forbes et al., 2018), and is increasingly found in patients with severe comorbidities (Woerther et al., 2017).

Frailty has been demonstrated to be superior to chronological age in assessing the risk of age-related adverse health outcomes. A frailty-associated co-abundance module composed of *Eggerthella*, *Coprobacillus*, and *Ruminococcus* was identified in community-dwelling adults (Maffei et al., 2017). A short-term prospective longitudinal cohort study observed that, with increasing frailty, residents had lower abundances of butyrate-producing bacterium, particularly *Clostridium* cluster XIVa and *Lachnospiraceae* (Haran et al., 2018). Gut microbiota diversity and composition were also altered in late elderly hospitalized patients (Zhang et al., 2020).

In a recent study, Lim et al. analyzed the relationship between gut microbiota and frailty in 176 Korean older adults and found that in older adults with frailty, the abundance of *P. copri* and *Coprococcus eutactus* were decreased, while that of the opportunistic pathogens *Bacteroides fragilis* and *Clostridium hathewayi* were increased (Lim et al., 2021). Notably, the relative abundance of the butyrate-producing *Coprococcus* is negatively associated with atopic disease, depression and irritable bowel syndrome (Lim et al., 2021), while *B. fragilis* toxin triggers a pro-carcinogenic, multistep inflammatory cascade in colonic epithelial cells (Chung et al., 2018). In addition, in a recent study of older patients with chronic kidney disease, the alpha diversity of the gut microbiota community was found to be similar between the control and frail groups, except that *Mogibacteriaceae, Coriobacteriaceae*, and *Eggerthella* spp were more abundant in the frail subjects (Margiotta et al., 2020). Some of these bacteria, which include *Coriobacteriaceae* and *Eggerthell*, are correlated with the occurrence of neurological and psychiatric disorders, such as multiple sclerosis and major depressive disorder (Fung et al., 2017).

Sarcopenia is considered a premonitory state for frailty. Picca et al. found that microbial alpha diversity did not significantly differ between physical frailty and sarcopenia (PF&S) and non-PF&S groups. However, their microbial composition was significantly different (Picca et al., 2019). In the PF&S group, *Oscillospira* and *Ruminococcus* were more abundant, whereas *Barnesiellaceae* and *Christensenellaceae* were less. *Ruminococcus* has been reported to accelerate the disease progression of amyotrophic lateral sclerosis in susceptible mice (Blacher et al., 2019). *Ruminococcus* and *Oscillospira*, which both belong to the order *Clostridiales*, are enriched in HIV-infected women (Wang et al., 2020). *Christensenellaceae*, which belongs to the bacterial phylum Firmicutes, is closely related to host health (Waters and Ley, 2019). The relative abundance of *Christensenellaceae* in the human gut is related to health status in multiple disease contexts, including metabolic disease and inflammatory bowel disease.

## Chronic Low-Grade Inflammation: A Driver of Frailty?

Chronic low-grade inflammation is a low-grade, systemic, unresolved smoldering chronic inflammatory state first proposed by Krabbe et al. in 2004 (Krabbe et al., 2004). It is part of a spectrum of immunosenescence characteristics (Franceschi et al., 2000), of which the chronic activation of the innate immune system is a prominent feature (Franceschi et al., 2018). Chronic inflammation is a crucial driver of metabolic disorders and the age-related decline of physical functions. It may also be a risk factor for some age-related diseases, such as cancer, depression, sarcopenia, and disability (Cesari et al., 2004; Grivennikov et al., 2010; Kohler et al., 2016). These age-related diseases have similar mechanisms as those underlying the development of frailty.

Sarcopenia is an age-related generalized skeletal muscle disorder characterized by decreased muscle mass and function. It is associated with a risk of adverse outcomes such as physical disability, low quality of life, and death, and it has strong links with physical frailty. Higher plasma concentrations of inflammatory biomarkers are associated with a decline in muscle mass and strength (Cesari et al., 2004). In contrast, long-term nonsteroidal anti-inflammatory drug (NSAID) use is linked to a decreased risk of muscle mass and loss of function (Landi et al., 2013).

More studies investigating the relationship between chronic low-grade inflammation and frailty may be helpful in developing scientific interventions. The mechanism may involve musculoskeletal metabolism and the endocrine and central nervous systems. Sarcopenia is considered the biological substrate for physical frailty and levels of the plasma inflammatory marker CRP are higher in patients with sarcopenia than in controls (Bano et al., 2017). Reduced muscle protein synthesis and increased muscle degradation are observed in pathological inflammation (Constantin et al., 2013; Wilson et al., 2017).

Chronic inflammation may also have adverse effects on the regenerative function of skeletal muscle satellite cells and can induce mitochondrial-mediated or death domain receptor-mediated apoptosis pathways in skeletal muscle cells (Jo et al., 2012; Walston, 2015). Insulin-like growth factor-1 (IGF-1) is related to many skeletal muscle anabolic pathways and can be used to delay the progression of muscle weakness. Under inflammatory conditions, it is maintained at relatively low levels. However, local expression of the *IGF-1ea* and *IGF‐1eb* transgenes in aged transgenic mice can alleviate age-related losses of muscle mass and strength loss (Ascenzi et al., 2019). During senescence, lower levels of sex steroids, growth hormones, and vitamin D are associated with increased baseline levels of inflammatory proteins (Hunt et al., 2010). In addition, chronic low-grade inflammation can further promote anorexia of aging and is associated with reduced mobility and impaired cognitive function, which contribute to the development of physical and cognitive frailty (Greco et al., 2019).

## How Do Gut Microbiota Cause Frailty?

### The Gut Microbiota, Inflammation, and Intestinal Permeability; the Gut-Muscle Axis; and the Gut-Brain Axis

**Figure 1:**
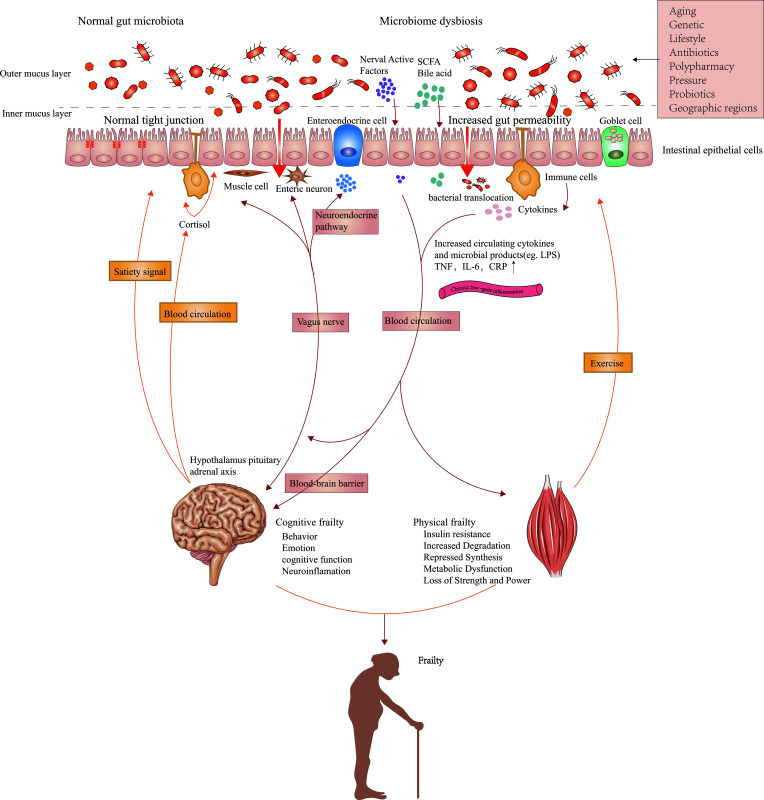
Possible mechanisms linking gut microbiota composition, chronic low-grade inflammation, and frailty. Impairments in the intestinal barrier caused by aged gut microbiota can promote the penetration and systemic dissemination of microbiota and its metabolites, which then leads to chronic low-grade inflammation by promoting the expression of proinflammatory factors. Metabolites from commensal microbiota as well as peripheral inflammation affect protein breakdown and synthesis in the muscle via multiple signaling pathways that result from inflammation and insulin sensitivity. The effects of muscles on gut microbiota are mainly realized by exercise. The brain controls the composition of gut microbiota by influencing peptides regulating satiety signals, intestinal function, and the secretion of neural chemicals. Intestinal dysbacteriosis affects inflammation and neurodegeneration in the central nervous system via immune, vagal, and neuroendocrine pathways, which ultimately lead to impaired cognitive function. Gut microbiota can also indirectly cause the onset of sarcopenia and physical frailty by affecting food intake.

The human gut microbiota is a complex and delicate commensal ecosystem affected by diet, metabolism, and immune function (Franceschi et al., 2018). The relationship between the host and healthy gut microbiota is mutually beneficial. The adaptability and plasticity of the gut microbiota allow them to affect the health and disease state of the host by adjusting immune and metabolic pathways. The aging-related remodeling of gut microbiota can facilitate the release of inflammatory products, programs diurnal rhythms in host metabolism (Kuang et al., 2019), and interact with other organs and tissues. We hypothesize that changes in the gut microbiome may play a role in the pathogenesis of physical frailty by regulating gut-derived chronic inflammation. We summarize the possible mechanisms in **Figure 1**.

### Increased Gut Permeability

Zonulin and fatty acid-binding protein-2, as paracellular integrity molecules of the intestinal epithelium, are biomarkers of gut permeability and pathophysiological epithelium integrity. Dysbacteriosis can upregulate these molecules (Stevens et al., 2018). Serum concentrations of zonulin and high mobility group protein B1 increase with age and are negatively correlated with critical indices of age-related frailty, such as skeletal muscle strength and habitual physical activity (Qi et al., 2017).

Aged gut microbiota display a reduced capacity to counteract adverse microbes or remove their metabolites, which contributes to the activation of inflammatory reactions and the induction of immune disorders (Zhao, 2010). Aging-associated microbiota can promote intestinal permeability and inflammation and eventually increase the levels of frailty-related pro-inflammatory cytokines such as IL-6 and TNF-α (Thevaranjan et al., 2017; Ticinesi et al., 2019). Fransen et al. transplanted gut microbiota from old conventional mice into young germ-free (GF) mice. They found that certain microbiota changes (i.e., lower levels of *Akkermansia* and higher levels of *TM7* bacteria and *Proteobacteria*) can contribute to inflammation in the small intestine and promote the leakage of inflammatory bacterial components into the circulation (Fransen et al., 2017). Studies in fruit flies have indicated that changes in microbiota precede and can predict aged-related intestinal barrier dysfunction and rapid health decline (Clark et al., 2015). Alterations in the microbiota that occur prior to intestinal barrier dysfunction are conductive to intestinal immune activation and modulate excretory function. In contrast, microbiota composition changes that occur after intestinal barrier dysfunction result in systemic immune activation and organismal death.

At the onset of solid food ingestion, the metabolites produced by the microbiota, such as butyrate, contribute to intestinal barrier maturation at the suckling-to-weaning transition. Gut-microbiota-derived metabolites have been suggested as a reasonable approach to maintain intestinal homeostasis (Beaumont et al., 2020). The most important gut microbial metabolites include short-chain fatty acids (SCFAs), amino acids, polyamines, bile acids, and lipids. Between these, SCFAs have been extensively studied and lower levels of SCFA are associated with age-related frailty (Nagpal et al., 2018). For example, the proportion of SCFAs-generating bacteria are decreased in nursing home residents with frailty (Haran et al., 2018). SCFAs can enhance intestinal barrier function by regulating tight junction proteins, including claudin-1, occludin, and zonula occludens-1. Additionally, it may induce hypoxia-inducible factor in intestinal epithelial cells and stimulate mucin secretion (Feng et al., 2018).

Intestinal dysbacteriosis contributes to increased lipopolysaccharide (LPS) expression, activation of the Toll-like receptor and degradation of the mucus layer, which lead to metabolic derangements. This process includes proinflammatory changes associated with intestinal barrier disruption such as alterations in the population of intestinal lamina propria cells and in the cytokine landscape. Bacteria or their products can enter the circulation through a damaged gut barrier. Some bacterial metabolites, such as phenylacetic acid, trimethylamine, or pathogen-associated-molecular-patterns, can promote chronic low-grade inflammation by inducing proinflammatory cytokines (Tilg et al., 2020).

### Changes in the Gut-Muscle Axis

Sarcopenia is an age-related generalized skeletal muscle disorder characterized by decreased muscle mass and function. It is associated with a risk of adverse outcomes such as physical disability, poor quality of life, or death, and it is strongly linked with physical frailty (Mijnarends et al., 2015; Picca et al., 2019). Sarcopenia and physical frailty have partially overlapping diagnostic criteria, such as loss of strength and decreased physical activity. Muscle mass depletion and the reduction of muscle function are the main indicators of physical frailty and sarcopenia. According to Mijnarends et al., sarcopenia is a central manifestation and a key component of frailty. Frail elderly have a 60% risk of suffering from sarcopenia (Mijnarends et al., 2015). Sarcopenia is considered the biological basis of physical frailty and is related to the physiopathologic pathways underlying the negative health-related outcomes of frailty (Landi et al., 2015).

Numerous experiments in animals indicate possible links between intestinal dysbacteriosis and muscle mass, muscle function, and physical performance. Lahiri *et al.* compared the skeletal muscles of GF mice with those of specific-pathogen-free (SPF) mice with a gut microbiota. GF mice displayed reduced muscle mass and strength, decreased IGF-1 expression, and reduced transcription of genes related to skeletal muscle growth and mitochondrial function. The transplantation of gut microbiota from SPF mice into GF mice improved skeletal muscle mass and the oxidative metabolic capacity of muscles (Lahiri et al., 2019), and SCFA supplementation reduced the muscle damage in GF mice. Clinically, few related studies are available, and those that do exist mainly use probiotic interventions. For example, Buigues et al. founded that compared with placebo, prebiotic administration greatly improved exhaustion and handgrip strength, which are two criteria for frailty (Buigues et al., 2016). Moreover, short-term synbiotic use can alter the gut microbiota composition and reduce pro-inflammatory cytokine TNF-α levels in older adults (Macfarlane et al., 2013).

The effects of muscles on the gut microbiota are mainly realized by exercise. Professional athletes have a higher diversity of gut microbiota (Clarke et al., 2014). Petersen et al. analyzed the gut microbiomes in competitive cyclists. They found that the time spent exercising per week was related to high abundances of *Prevotella*, which was correlated with a number of amino acid and carbohydrate metabolism pathways (Petersen et al., 2017). Exercise induces changes in gut microbiota composition and metabolic capacity, and these changes are contingent on obesity status. However, excessive training may contribute to dysfunction of the intestinal mucosa, which increases and decreases, respectively, the tendencies for opportunistic pathogens and anti-inflammatory bacteria (Karl et al., 2017). The effects of exercise on gut microbiota have been corroborated by animal experiments. The changes in the gut microbiota following exercise is conducive to the suppression of gut inflammation and an increase in energy harvest (Lamoureux et al., 2017; Allen et al., 2018).

The effects of an altered gut microbiota on muscle mass and function may be mediated by potential mechanisms involving metabolites or the endocrine or immune systems. The exposure of GF mice to microbiota can increase both bone formation and resorption (Yan et al., 2016). This process is accompanied by increases in IGF-1, a survival factor that is known to affect the growth and regeneration of skeletal muscle (Yan et al., 2016). One strain of *Escherichia coli* has been observed to prevent muscle wasting in mice with infections or physical damage to the intestine by acting on IGF-1 signaling in skeletal muscle; this effect depends on the NLRC4 inflammasome (Palaferri Schieber et al., 2015). Ghrelin is essential for the maintenance of aging muscles. In old ghrelin-deficient mice, fasting-induced muscle loss is exacerbated. The absence of ghrelin is associated with a pro-inflammatory microbiome profile, which is manifested in reduced levels of the butyrate-producing bacteria *Roseburia* and *Clostridium* cluster XIV*b* (Wu et al., 2020). Under conditions of adaptive immune deficiency, gut microbiota can promote a change from a pro-metabolic to a pro-immune phenotype by increasing interferon-related genes and suppressing metabolic genes related to lipid absorption. These changes ultimately lead to muscle wastage and weight loss (Chassaing et al., 2012).

Changes in gut microbiota can influence the inflammatory state of the body. More specifically, chronic low-grade inflammation can affect protein breakdown and synthesis in muscle *via* various signaling pathways (e.g., the ubiquitin-proteasome pathway, calpains, autophagy pathway, and apoptosis), which then lead to losses in muscle quality, strength, and function (Dalle et al., 2017). Increases in TNF-α, IL-6, and fatty free acids seem to contribute to skeletal muscle proteolysis and insulin resistance, two main factors in several forms of muscle atrophy (Xia et al., 2017). In old rats, the use of NSAIDs increases muscle protein synthesis and decreases proteolysis (Rieu et al., 2009). Therefore, the control of intestinal-originating chronic low-grade inflammation may be therapeutic in preventing sarcopenia and limiting muscle strength loss (Przewlocka et al., 2020).

Obesity status, exercise modality, and exercise intensity may mediate the gut microbiota changes that are induced by exercise, which are largely unrelated to diet. Mailing et al. summarized the potential mechanisms underlying the effect of exercise on gut microbiota, which mainly involve ischemia, heat stress, metabolic flux, gut barrier, mucus layer, gut motility, mechanical forces, the vagus nerve/enteric nervous system, hormones/myokines, and bile acids. Some believe that intestinal immunity plays an important role in this process (Mailing LJ et al., 2019). Moderate-intensity exercise may improve intestinal immunity by increasing lymphocyte proliferation (Valdes-Ramos et al., 2010). Further, γδ intraepithelial lymphocytes of the small intestine are critical mediators in the homeostasis between host and gut microbiota at the intestinal mucosal surface (Ismail et al., 2011). High-intensity exercise can result in brief intestinal permeability and increase the contact between gut microbiota and the immune system, which can then impact gut microbial communities.

### Changes in the Gut-Brain Axis

The gut-brain axis is a duplex information system linking the intestine with the central nervous system (CNS). Stress signals from the brain can impact gastrointestinal function and gut microbiota composition *via* the efferent nerves and the hypothalamic-pituitary-adrenal (HPA) axis (Cox and Weiner, 2018). The continuous activation of the HPA axis increases the secretion of cortisol, which can then regulate the neuro immune signal response and affect the integrity of the intestinal barrier (Morais et al., 2021). Conversely, the effect of the gut microbiota on the brain mainly involves neural, endocrine, and immune pathways. Gut microbiota can directly or indirectly affect the nervous system by producing metabolites, acting on the neuroendocrine system, and regulating neurotransmitter concentrations. Gut microbiota and its metabolites can also affect the brain and thereby behavior by acting on the vagus nerve and enteric nervous system (Morais et al., 2021).

Dysregulation of the microbiota-gut-brain axis is involved in the pathophysiology of several neurological disorders, such as Alzheimer’s disease (AD), autism spectrum disorder, brain injury, multiple sclerosis, and stroke (Cryan et al., 2020). Many clinical and animal studies confirm the link between the two-way communication between the gut microbiota and the brain with cognitive impairment. In human studies, the changes in the gut microbiota in patients with mild cognitive impairment are similar to those of AD, suggesting that the gut microbiota are involved in the early pathogenesis of AD (Li et al., 2019). A model based on specific bacteria and SCFAs can accurately predict post-stroke cognitive impairments after stroke onset (Liu et al., 2020). Therapeutic strategies of gut microbiota regulation such as probiotics or fecal microbiota transplantation can be an effective way to prevent or treat cognitive impairment (Sun et al., 2020).

In animal studies, traumatic brain injury in mice changes the gastrointestinal tract as indicated by increased intestinal permeability and bacterial translocation (Ma et al., 2017). Older mice exhibit spatial memory deficits and increased anxiety-like behaviors relative to younger mice, and they also have increased gut permeability and levels of peripheral pro-inflammatory cytokines. Age-related alterations in the microbiota (e.g., the phylum *TM7*, the family *Porphyromonadaceae*, and the genus *Odoribacter*) are similar to those in many inflammatory diseases. From these, *Porphyromonadaceae* is relevant to cognitive impairment and affective disorders. Scott et al. proposed that changes in gut microbiota that may be involved in behavior impairment and affective and cognitive functions are also involved in gut permeability and peripheral inflammation (Scott et al., 2017). Moreover, probiotics supplementation may delay the onset of aging-related cognitive deficits (Yang et al., 2020).

Changes in gut barrier permeability and the blood-brain barrier affect the gut-brain axis (Osadchiy et al., 2019). In the drosophila dementia model, intestinal microflora dysbiosis is correlated to tumor necrosis factor- and c-Jun N-terminal kinase (JNK)-mediated inflammatory deterioration as well as to neurodegenerative brain pathways (Wu et al., 2017). Increased JNK activity can cause intestinal barrier dysfunction and microbial dysbiosis in the drosophila intestinal tumor model and mediate inflammatory deterioration *via* dysplasia of the drosophila midgut (Zhou and Boutros, 2020; Tamamouna et al., 2020). Peripheral inflammation can also lead to impaired cognitive function by directly affecting neuroimmune processes in the CNS. In an aging AD mouse model, changes in gut microbiota composition stimulate neuroinflammation by promoting the accumulation of phenylalanine and isoleucine (Wang et al., 2019). The metabolites of dietary tryptophan, which are controlled by commensal microbiota, can directly act on microglia and astrocytes in the CNS *via* the aryl hydrocarbon receptor to control inflammation and neurodegeneration (Rothhammer et al., 2018). The decreases in SCFAs produced by gut microbiota can potentially reduce amyloid deposition in both the brain and the gut (Zhang et al., 2017).

Certain bacterial components and metabolites that depend on bacterial growth cycles can stimulate intestinal satiety pathways (Fetissov, 2017). A review by Fetissov et al. proposed an integrative model of appetite control. According to this model, the short- and long-term regulation of appetite may involve the integration of signals from both the host and the bacteria. In animals, high doses of LPS reduce food intake. LPS can activate intracellular signaling pathways, increase levels of the pro-inflammatory mediator IL-1β, and further induce a chronic inflammatory state that includes hypothalamic inflammation. This process is similar to the expression pattern of neuropeptides in obesity, anorexia nervosa, and cachexia. Altogether, these findings suggest that gut microbiota can indirectly induce sarcopenia and physical frailty in older patients by promoting malnutrition (Ticinesi et al., 2019).

## Conclusions and Future Perspectives

Studies have established that inflammation, characterized by elevated levels of blood inflammatory markers, contributes to the development of frailty in older adults. Alterations in gut microbiota composition and its metabolites may be key regulators in mediating chronic inflammation in frailty. However, animal models of frailty are in its early phases, and the empirical data is lacking. The present studies on gut microbiota and frailty are mainly clinical cross-sectional studies. Genetics, age, gender, nutrition intake, lifestyle, polypharmacy, social vulnerability, and geographic regions may all influence gut microbiota composition and function. Consequently, personalized regulation based on gut microbiota is necessary. Developing a verifiable predictive model for gut microbiota and frailty in older adults *via* large scale longitudinal research may help in determining the causal relationship between microbiota and disease, as well as in its early diagnosis and intervention.

## Author Contributions

Data analyses and wrote the manuscript: YX, DC, MW, and XJ. Manuscript preparation: XJL and XXL. Study design, project management, financial support, and manuscript revision: ZX. All authors contributed to the article and approved the submitted version.

## Funding

This study was supported by the National Key Research and Development Program of China (2018YFC2002000).

## Conflict of Interest

The authors declare that the research was conducted in the absence of any commercial or financial relationships that could be construed as a potential conflict of interest.
